# Eustachian valve endocarditis: a rare localization of right side endocarditis. A case report and review of the literature

**DOI:** 10.1186/1476-7120-3-30

**Published:** 2005-09-19

**Authors:** Adriano M Pellicelli, Paolo Pino, Antonio Terranova, Cecilia D'Ambrosio, Fabrizio Soccorsi

**Affiliations:** 1 Consultant of Infectious disease, San Camillo Forlanini Hospital, via Portuense 332 00149 Rome Italy; 2 Department of Cardiovascular Science San Camillo Forlanini Hospital via Portuense 332 00149 Rome Italy; 3 Department of Internal Medicine San Camillo Forlanini Hospital via Portuense 332 00149 Rome Italy

**Keywords:** Eustachian valve endocarditis, transthoracic echocardiography, intravenous drug abuse, staphylococcus aureus

## Abstract

**Background:**

Right-sided endocarditis occurs predominantly in intravenous drug users, in patients with pacemaker or central venous lines and in patients with congenital heart disease. The vast majority of cases involve the tricuspid valve. Eustachian valve endocarditis is an uncommon disease with similar signs and symptoms of the tricuspid valve endocarditis. A series of only 16 cases of eustachian valve endocarditis are reported in the literature.

**Case Presentation:**

We present a case of a 25-year old woman with intravenous drug abuse who had a staphylococcus aureus tricuspid valve endocarditis associated to eustachian valve endocarditis. Transthoracic echocardiography, as first line examination, showed the vegetations on tricuspid and eustachian valve.

**Conclusion:**

Our case describe an unusual location of right side endocarditis in a intravenous drug abuser. In our case, in accord with other cases described in the literature, transthoracic echocardiography disclosed eustachian valve endocarditis.

Antimicrobial management is not altered by the recognition of eustachian valve endocarditis. Antibiotic treatment and duration of eustachian endocarditis depends on the isolated organism and is similar to antibiotic therapy used in native valve endocarditis.

## Background

Right-sided endocarditis occurs predominantly in intravenous drug abusers (IVDA), in patients with pacemaker or central venous lines and in patients with congenital heart disease. The vast majority of cases involve the tricuspid valve. In the case that we describe, tricuspid valve endocarditis was associated to eustachian valve endocarditis (EVE). Eustachian valve is a structure localized between the inferior vena cava and the right atrium. In the fetal circulation this valve directs the blood flow from the vena cava through the foramen oval into the left atrium. In an adult the persistence of this valve is uncommon. We report the 12th reported case of infection causing endocarditis of the eustachian valve, and we present a review of the existing English medical literature.

## Case Report

**Figure 1:**
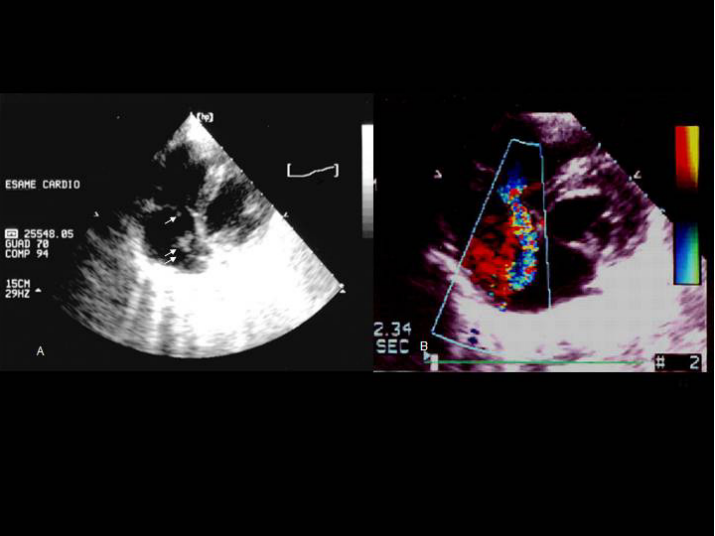
A, Two dimensional transthoracic echocardiogram (modified 4 chambers view) showing a vegetation (2.5 cm × 1.3 cm) on eustachian valve (double arrow) and another small vegetation (0.5 × 0.6 cm) on tricuspid valve (single arrow). B, Color flow doppler echocardiogram (modified 4 chambers view) showing a moderate tricuspid regurgitation with flow toward the eustachian valve.

**Figure 2:**
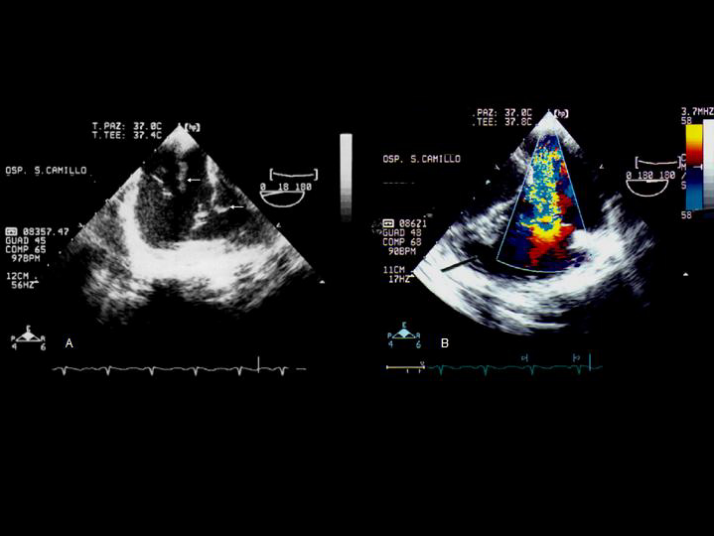
A Transesophageal echocardiographic view showing two peduncolated vegetations attached to the eustachian valve and another vegetation on tricuspid valve. B, Color flow doppler echocardiogram which confirms a moderate tricuspid regurgitation with flow toward the eustachian valve.

A 25 year old woman with a seven years history of intravenous drug abuse was admitted to our hospital after 6 days with fever and chills. Her vital signs included a temperature of 39°C, a heart rate of 110 bpm, a blood pressure of 125/70 mmHg. Findings on cardiac examination were unremarkable, as were the results of the remainder of the evaluation. Results of laboratory examinations included a white blood cell count of 12.000 cells/mm3, an erythrocyte sedimentation time of 100 mm and a reactive C protein of 5 mg/100 ml (0.5 upper limit). A set of three peripheral blood culture from different sites were made. A Transthoracic echocardiography (TTE) was performed the same day as the patient was admitted to the hospital. A structure due to an eustachian valve was noted in the right atrium. Furthermore a mobile mass of 1.5 cm × 0.8 cm was noted to be attached to the eustachian valve. Another mobile mass of 0.6 cm × 0.8 cm was noted on tricuspid valve (Figure 1). A diagnosis of endocarditis on eustachian and tricuspid valve was made. The patient started an empiric antibiotic therapy with oxacillin 12 gr/day ev and netilmicin 300 mg/day ev. After 5 days from admission in the hospital, 3 blood cultures grew methicillin susceptible staphylococcus aureus. After eight days of persistence of fever a transesophageal echocardiography (TEE) was performed to exclude local complication related to infective endocarditis. The TEE confirmed the diagnosis of eustachian and tricuspid valve endocarditis and excluded local complications due to infective endocarditis (Figure 2). In the following days the patient became afebrile and was discharged form the hospital in good clinical condition after a 6 weeks course of antibiotic therapy.

## Conclusion

A review of the literature through medline revealed only 16 other cases of EVE, which are summarized in the Table.

In the cases reported in the literature, IVDA was the most common risk factor for EVE. In all IVDA patients with EVE, staphylococcus aureus was the only pathogenetic organism isolated, while in the remaining cases different organisms were isolated. As described by Frontera et al., staphylococcus aureus was the most common pathogen in right heart endocarditis among IVDA [10]. In IVDA there is an increased right side expression of matrix molecules capable of binding microorganisms, these molecules (as serum fibronectin and fibrinogen) are known to rapidly coat foreign surfaces as catheters, but may also coat particulate matters that are injected as contaminants in IVDA. In particular staphylococcus aureus possesses surface proteins that enable it to adhere to these molecules. This can partially explain why staphylococcus aureus is the most common isolated organism in IVDA. The frequent localization of this organism in right-side infection could be due to a large directly injected bacterial load in these patients. In the adult, the eustachian valve when present, directs the blood flow from inferior vena cava into the right atrium. In our case it is possible that particular matters derived from continuous intravenous drug use could have damaged the eustachian valve over time, but it could also be hypothesized that a flow of insufficiency directed from the infected tricuspid valve to eustachian valve could have been responsible for a damage of the eustachian valve and of a subsequent implant of bacterial organisms derived from tricuspid valve. Because the eustachian valve was frequently the sole focus of intracardiac infection in the cases reported in the literature, it seems more reasonable to think to the first pathogenetic hypothesis. Over 16 cases described in the literature, in 10 cases diagnosis was made by TEE. Transesophageal echocardiography is not indicated as initial examination in the diagnosis of native valve endocarditis. According to American College Cardiology/American Heart Association (ACC/AHA) guidelines for the clinical application of echocardiography [11], when the valvular structure or pathology is well visualized by TTE, there is no indication to perform TEE. In the four cases described by Sawhney et al., TTE results were normal and the indication to perform a TEE derived from a strong clinical suspicion of bacterial endocarditis in the presence of a bacteremia [6]. In the case described by Bowers et al. several vegetation was noted in different places by the TTE on the tricuspid valve but no eustachian valve was seen. In this case TEE was used as a revaluation study in the presence of a worsening in the clinical status of the patient [7]. The diagnosis of EVE was occasional and the treatment of the solely tricuspid endocarditis would not have changed the prognosis of the patient. In the case described by Georgeson et al. TTE was not diagnostic although TEE revealed a large, pedunculated, and highly mobile vegetation attached to the eustachian valve [3]. In the study of San Roman et al. [9] TEE did not add any new information to that obtained by TTE in 5 cases of eustachian valve endocarditis. They stated that both techniques offer similar information although their patients were young and had an excellent acoustic windows. In our case TTE revealed mobile vegetations on eustachian and tricuspid valve and TEE was performed only to exclude local complications related to infective endocarditis.

## Competing interests

All the Authors disclose any financial competing interests but also any non-financial competing interests that may cause them embarrassment were they to become public after the publication of the manuscript.

## Authors' contributions

AMP performed transthoracic echocardiographic study, attended the patient and drafted the manuscript, PP performed Transesophageal echocardiographic study and drafted the manuscript, AT drafted the manuscript, CD attended the patient and collected all the data, FS drafted the manuscript.

**Table 1:** Previously reported cases of eustachian endocarditis

**Ref.n°**	**Time of diagnosis**	**Valve involved**	**Source of infection**	**Isolated organism**	**Method of diagnosis**
1	Postmortem	eustachian and mitral valve	Pneumoniae	S. aureus	Autopsy
2	Antemortem	eustachian valve	IVDA	S. aureus	TEE
3	Antemortem	eustachian valve	IVDA	S. aureus	TEE
4	Antemortem	eustachian valve	IVDA	S. aureus	TEE
5	Antemortem	eustachian valve	Infected PMK	Enterobacter cloacae	TEE
6	Antemortem	eustachian valve	IVDA	S. aureus	TEE
6	Antemortem	eustachian valve	IVDA	S. aureus	TEE
6	Antemortem	eustachian valve	Hemodialysis	E. coli	TEE
6	Antemortem	eustachian valve	Hemodialysis	Proteus vulgaris	TEE
7	Antemortem	eustachian and tricuspid valve	IVDA	S. aureus	TEE
8	Antemortem	eustachian valve	none	Streptococcus viridans	TEE
9	Antemortem	eustachian, mitral and tricuspid valve	IVDA	S. aureus	TTE
9	Antemortem	eustachian, tricuspid valve	IVDA	S. aureus	TTE
9	Antemortem	eustachian, tricuspid pulmonary and mitral	LH and central venous line	S. aureus	TTE
9	Antemortem	eustachian, tricuspid and central venous line	LH and central venous line	S. aureus	TTE
9	Antemortem	eustachian valve	IVDA	S. aureus	TTE
Case report	Antemortem	eustachian valve and tricuspid valve	IVDA	S. aureus	TTE

Ref. (references number); IVDA (intravenous drug abuse); TEE (Transesophageal echocardiography); TTE (Transthoracic echocardiography); PMK (Pacemaker); S. aureus (staphylococcus aureus)
